# Passing Away

**Published:** 1858-02

**Authors:** 


### PASSING AWAY.

Of all the multitudes who served in the army of the Revolution, only three hundred and forty-six pensioners remained on the last Fourth of July.

Ten per cent of all the Revolutionary pensioners died last year. But the hardy old soldiers must have felt themselves endowed with a new youth, when they came home from the wars, and taken to themselves young wives; for there are ten widows of soldiers living where there is one soldier, or four thousand seven hundred and two to three hundred and forty-six.

